# Autophagy and Its Lineage-Specific Roles in the Hematopoietic System

**DOI:** 10.1155/2023/8257217

**Published:** 2023-05-03

**Authors:** Kazi Md Mahmudul Hasan, Md Anwarul Haque

**Affiliations:** ^1^Department of Health Technology and Informatics, The Hong Kong Polytechnic University, Hung Hom, Kowloon, Hong Kong; ^2^Department of Biotechnology and Genetic Engineering, Islamic University, Kushtia 7003, Bangladesh; ^3^Department of Neurology, David Geffen School of Medicine, The University of California, 710 Westwood Plaza, Los Angeles, CA 90095, USA

## Abstract

Autophagy is a dynamic process that regulates the selective and nonselective degradation of cytoplasmic components, such as damaged organelles and protein aggregates inside lysosomes to maintain tissue homeostasis. Different types of autophagy including macroautophagy, microautophagy, and chaperon-mediated autophagy (CMA) have been implicated in a variety of pathological conditions, such as cancer, aging, neurodegeneration, and developmental disorders. Furthermore, the molecular mechanism and biological functions of autophagy have been extensively studied in vertebrate hematopoiesis and human blood malignancies. In recent years, the hematopoietic lineage-specific roles of different autophagy-related (*ATG*) genes have gained more attention. The evolution of gene-editing technology and the easy access nature of hematopoietic stem cells (HSCs), hematopoietic progenitors, and precursor cells have facilitated the autophagy research to better understand how *ATG* genes function in the hematopoietic system. Taking advantage of the gene-editing platform, this review has summarized the roles of different *ATG*s at the hematopoietic cell level, their dysregulation, and pathological consequences throughout hematopoiesis.

## 1. Autophagy

The hallmark of autophagy is the cellular proteins and organelle clearance through lysosomal degradation and recycling of cytoplasmic materials, such as lipids, proteins, and organelles into smaller forms and release of amino acids, nucleotides, and fatty acids to maintain cellular homeostasis [1]. Various stress stimuli including oxidative stress, redox stress, endoplasmic reticulum (ER) stress, nutrient deprivation, mitochondrial damage, and hypoxia are responsible for inducing autophagy [2]. Autophagy machinery is orchestrated by the autophagy-related (*ATG*) genes, and till now, approximately 40 *ATG* genes have been identified in *Saccharomyces cerevisiae* yeast. In yeast, autophagy is initiated in response to various intracellular and extracellular stimulating factors, whereas ATG13 anchors unc-51 like autophagy-activating kinase 1 (ULK1) to form a cup-shaped preautophagosomal structure (PAS) [3], and subsequently, Atg proteins including Atg2 and Atg18 get localized to initiate autophagosome biogenesis [4, 5]. Most of the yeast *ATG* genes have mammalian counterparts which regulate various steps of the autophagy pathway [6]. Mammalian autophagy initiation is associated with the formation of a cup-like or omega-shaped (Ω) lipid bilayer membrane, also known as an “omegasome” near the endoplasmic reticulum (ER). Omegasomes further colocalize with phosphatidylinositol 3-phosphate (PI(3)P) and autophagosomal marker microtubule-associated protein light chain 3 (MAP-LC3) [7]. Therefore, at the initiation of PAS or omegasome, phagophores increase in size and elongate into cup-shaped structures before engulfing the cellular materials. Apart from yeast and mammals including *Mus musculus*, there are nematodes such as *Caenorhabditis elegans* and insects such as *Drosophila melanogaster* and *Bombyx mori*, where *ATG* genes have been identified [8–10]. Due to the diverse regulatory functions of *ATG* genes in metabolic homeostasis, autophagy plays an essential role in maintaining cellular development, and its dysregulations are associated with mammalian aging, developmental defects, neurodegeneration, and cancer [11–14].

To interpret autophagy more efficiently, measuring autophagy flux is critical, as it indicates whether autophagy cargos have been successfully degraded at the end of the autophagy process. The term “autophagic flux” refers to the entire autophagic process, which includes the formation, maturation, fusion, and disintegration of autophagosomes with lysosomes as well as the release of macromolecules recycled back into the cytosol. However, under nutrient-rich conditions, autophagy does not affect metabolic flux [15]. Flux can be quantitatively measured by LC3-II turnover using immunoblot analysis in the presence and absence of lysosomal inhibitors, such as chloroquine, bafilomycin A1, and NH_4_Cl [16]. Furthermore, an autophagic flux report system has been developed, which combines LC3 with the acid-resistant mCherry and the acid-sensitive GFP to measure autophagosome maturation and degradation. In this system, both LC3 with red fluorescent mCherry and green fluorescent GFP is fused to form yellow puncta and detected in autophagosomes [17]. Due to GFP degradation by acid lysosomal proteases, the green fluorescence of autophagosomes is lost when autophagosomes fuse with lysosomes, which results in LC3 emitting only red fluorescence. This dynamic switch indicates an active autophagic flux process when yellow fluorescence is switched to red. Additionally, p62 accumulation can be used as an autophagy flux indicator [18].

## 2. Hematopoiesis

Hematopoiesis is the process by which the multipotent hematopoietic stem cells (HSCs) give rise to all blood cell lineages such as myeloid cells, leukocytes, erythrocytes, and platelets through the generation of progenitor cells [19]. These progenitor cells further undergo gradual fate restriction and are eventually identified as mature blood cells. Physiologically, all these blood cells reside inside the bone marrow microenvironment known as the HSC niche [20]. In mammals, hematopoiesis occurs sequentially in four distinct areas including the yolk sac, aorta-gonad mesonephros (AGM), fetal liver, and bone marrow [19]. The first wave of blood cell production inside the yolk sac is called “primitive.” Primitive hematopoiesis generates red blood cells for tissue oxygenation as the embryo grows quickly. Hematopoiesis in the primitive state is transient and is quickly replaced by adult-type “definitive” hematopoiesis. Throughout embryogenesis, definitive hematopoiesis is associated with hematopoietic stem cells and multipotent progenitors (HSCs/MPPs), which later become mature blood cells and immune cells [21]. Normal hematopoiesis maintains a unique balance between cellular differentiation, quiescence, and self-renewal capacity of hematopoietic stem and progenitor cells (HSPCs).

## 3. Different Forms of Autophagy in Hematopoiesis

**Figure 1:**
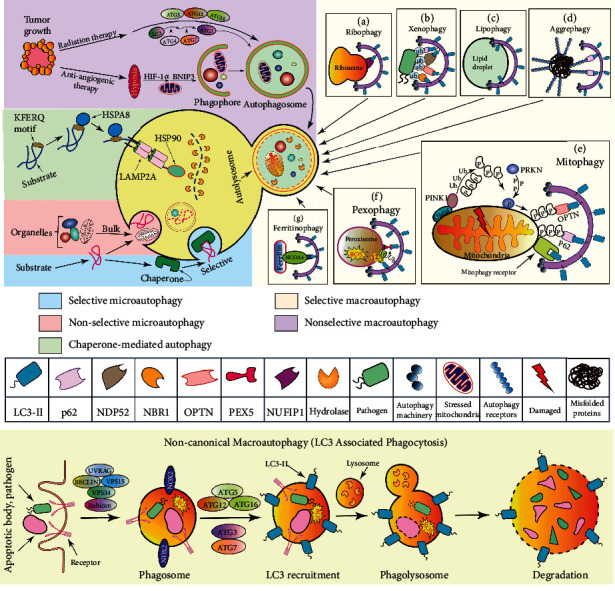
Schematic representation of the different types of autophagy. Selective macroautophagy is a strictly regulated cytosolic cargo degradation pathway for the removal of excess ribosomes—ribophagy (a), intracellular pathogens—xenophagy (b), lipid droplets—lipophagy (c), superfluous protein aggregates—aggrephagy (d), polluted mitochondria—mitophagy (e), dispensable peroxisomes—pexophagy (f), and ferritin iron—ferritinophagy (g). Under stress, tumorigenesis, anticancer therapy, and nonselective macroautophagy are initiated with the isolation of the phagophore membrane, autophagosome formation, maturation, and degradation in the lysosome. Likewise, macroautophagy and microautophagy can be selective, while most microautophagy is the nonselective and bulk degradation of cargo molecules. Chaperone-mediated autophagy (CMA) is the translocation of the motif-bearing substrate molecules to the lysosomal membrane, whereas the lysosomal hydrolases degraded the proteins and release amino acids. During LAP, a common form of noncanonical macroautophagy, ATGs from the PIK3 complex, ATG5-ATG12, and LC3 conjugation system incorporated in phagolysosome formation and its degradation without the ULK1 complex autophagy.

**Figure 2:**
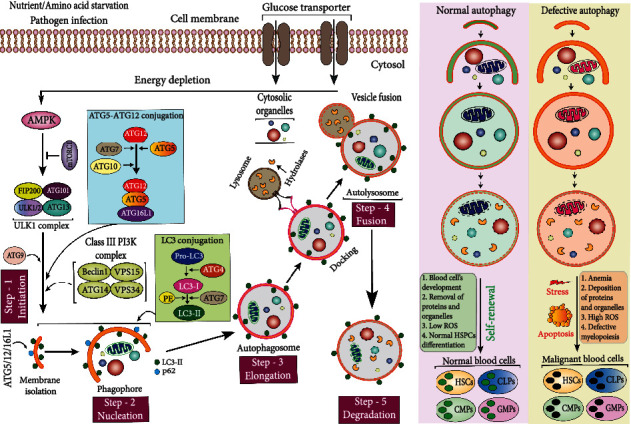
Macroautophagy and its role in hematopoiesis. During macroautophagy, AMPK activates the ULK1 complex and consequently induces the membrane isolation process where PI3Ks and ATG9 take part as positive regulators and enable autophagosome formation with the aid of ATG12-ATG5 and the LC3 conjugation systems. The ATG5–ATG12–ATG16L1 complex induces LC3 conjugation, whereas LC3 is cleaved by ATG4 protease to form LC3-I, and cytosolic LC3-1 is further conjugated with PE to form LC3-II. Afterward, autophagosomes come in contact with the lysosome which has the hydrolase enzymatic activity to fuse with autophagosome to form autolysosome for cytosolic cargo degradation. The normal state of autophagic activity is essential for the maintenance of blood cell homeostasis. Defective autophagy results in imbalanced hematopoiesis and impeded HSCs' self-renewal, leading to the generation of malignant blood cell types.

Macroautophagy, the most thoroughly researched form of autophagy, hereafter referred to as autophagy is the cellular proteins and organelle clearance mechanism through lysosomal degradation. Autophagy has been previously described as a nonselective or canonical nutrient recycling phenomenon that occurs ceaselessly at the basal level and delineates the random consumption as well as successive degradation of cytoplasmic organelles, pathogens, and aggregated proteins [1]. In nonselective macroautophagy, various external stimuli including pathogen infection, nutrient deprivation, and radiation therapy enhance autophagy by upregulating autophagy mediators such as *ATG3*, *ATG5*, *ATG12*, and *ATG16L1* (Figure 1). During hypoxia-mediated nonselective macroautophagy, hypoxia-inducible factor-1*α* (HIF-1*α*) upregulates the expression of mitophagy marker BNIP3 (BCL2/adenovirus E1B 19 kDa-interacting protein 3) [22]. A detailed overview of the nonselective or canonical macroautophagy is narrated in Figure 2.

In selective autophagy, different autophagy receptors play critical roles in regulating cellular homeostasis by facilitating the turnover of proteins, aggregates, organelles, and pathogens via lysosomal recycling. As hematopoietic cells need to meet their specific intracellular turnover requirements, the evolutionary conserved selective degradation pathways are increasingly being recognized for their importance to orchestrate macromolecular complexes and organelles intracellularly. In ribophagy, nuclear FMR1-interacting protein 1 (NUFIP1) selectively targets ribosomes to the phagophore (Figure 1(a)) [23], while in xenophagy, pathogen-containing vacuole disruption leads to xenophagy, while pathogens are targeted by ubiquitin (Ub) ligases, following autophagy adaptor protein recruitment including nuclear dot protein 52 kDa (NDP52), a neighbor of BRCA1 gene 1 (NBR1), optineurin (OPTN), and p62 that binds LC3 on the phagophore membrane (Figure 1(b)) [24–27]. However, the biological functions and molecular mechanisms of both xenophagy and ribophagy in hematopoietic cells are yet to be elucidated. Another selective autophagy is lipophagy which includes phagophore formation and expansion on lipid droplets (Figure 1(c)) [28]. During starvation and nutrient stress, lipophagy releases fatty acids and other lipid molecules from lipid droplets and maintains HSCs and myeloid cell biology via lipoprotein lipase (LPL) mediated lipid catabolism [29, 30]. It has been indicated that LPL activity is essential to regulate definitive hematopoiesis. When LPL activity abrogated, HSC markers *Runx1* and *Cmyb*, erythrocyte marker *Betaglobin*, and lymphocyte marker *Rag1* expression have been diminished, which indicates the erythropoietic and lymphopoietic defects as well as hyperlipidemia [29]. In aggrephagy, aggregated proteins become ubiquitinated and selected for autophagic degradation by ubiquitin-binding autophagy receptors such as p62, NBR1, OPTN, NDP52, and Tollip (Figure 1(d)) [31]. However, the role of aggrephagy in hematopoiesis and leukemia remains poorly understood. There has been evidence implicating aggrephagy in autophagic degradation of aggregated oncoproteins in acute leukemia, such as promyelocytic leukemia (PML) retinoic acid receptor alpha (RARA) fusion protein degradation via p62-mediated aggrephagy [32]. A more common and widely studied form of selective autophagy is mitophagy. During mitophagy, mitochondria are selectively degraded by PTEN-induced kinase 1 (PINK1) dependent pathway and receptor-mediated mechanisms [33]. In PINK1-dependent mitophagy, PINK1 phosphorylates ubiquitin, and PRKN promotes outer mitochondrial membrane (OMM) protein ubiquitination. Ubiquitinated proteins are then linked to ubiquitin-binding autophagy receptors including optineurin (OPTN) and p62 that subsequently interact with LC3-II, triggering the degradation of mitochondria (Figure 1(e)) [34]. Most HSPCs are in a quiescent state at basal level in the bone marrow (BM) stem cell niche, and PINK1-dependent mitophagy plays important roles in maintaining hematopoietic niche, as well as quiescence, stress response, and self-renewal of HSPCs [35]. HSC quiescence and stemness are also maintained through mitophagy during aging, which actively removes healthy mitochondria to preserve HSC regenerative capacity [36]. Mice with conditional deletion of *Atg12* have shown active mitochondrial mass, increased ROS, loss of quiescence and promyeloid differentiation, and hyperactive oxidative metabolism in HSCs. Further treatment with autophagy inhibitor bafilomycin A1 (BafA1) has exacerbated the condition in aged HSCs, indicating an *Atg12* autophagy-dependent role of mitochondria in HSC homeostasis [36]. Furthermore, mitophagy is also essential for the self-renewal and expansion of HSCs [37]. Ito et al. have used *Tie2*-GFP^+^ HSCs as HSC markers, while their gene expression data have shown a higher expression of mitophagy-related genes, including *Pink1*, *Parkin* (*Park2*), *Map1lc3a* (*Lc3*), *Optineurin*, and *p62/Sqstm1* in *Tie2*-GFP^+^ HSCs. Additionally, they found that silencing mitophagy via *Park2* or *Pink1* suppression not only eliminated Tie2-GFP^+^ cell growth but also inhibited the HSC cell maintenance, indicating that mitochondrial clearance is critical to maintaining HSC stemness by inducing mitophagosome formation [37]. Most of these genes were further upregulated by the activation of the PPAR-FAO pathway. However, mitophagy plays a paradoxical role in AML cells and normal hematopoietic cells. An increased level of mitophagy has been documented in AML cells, whereas macroautophagy inhibitors, like chloroquine (CQ), Lys05, and bafilomycin A1, are believed to decrease mitophagy in AML cells and increase antileukemic effects under hypoxia [38]. The results of this study open up a new therapeutic avenue for autophagy inhibitors in the treatment of AML. Selective degradation of peroxisomes refers to as pexophagy. A key component of mammalian pexophagy is the ubiquitination of peroxisomal proteins and the recognition of those proteins by SQSTM1 and NBR1 [39, 40]. Several studies identified the peroxisomal biogenesis factor 5 (PEX5) monoubiquitination as a cargo signal for peroxisome degradation [41, 42]. During pexophagy, PEX5 phosphorylation also induces PEX2-PEX10-PEX12 E3 ligase-mediated ubiquitination and Ub–PEX5 recognition (Figure 1(f)) [43]. Pexophagy can regulate mesenchymal stem cell aging in the bone marrow by inducing PEX10 and PEX14 activity through endothelial PAS domain-containing protein 1 (Epas1) regulation and suppressing ROS levels [44]. Pexophagy may also play a critical role in regulating monocyte biology [45]. As reported by Zhang et al., the PEX5 peroxisome import receptor binds serine/threonine kinase ataxia-telangiectasia mutated (ATM) and localizes it to peroxisomes. ATM kinase is activated by ROS which further phosphorylates PEX5 and subsequently monoubiquitinates it [46]. There is evidence that some widely used DNA-damaging agents in AML clinical treatment, such as doxorubicin, mitoxantrone, and etoposide, induced DNA damage response and ATM activation, which indicates that these agents could trigger pexophagy [47]. Further research is needed to determine whether pexophagy affects AML progression and therapeutic response. Ferritinophagy plays a critical role in the production and maintenance of erythrocytes and involves the iron-dependent ferritin (an iron-storing protein) breakdown. In ferritinophagy, ubiquitinated nuclear receptor coactivator 4 (NCOA4) selectively mediates the degradation of ferritin by targeting ferritin to LC3-II (Figure 1(g)) [48]. Using the zebrafish model and cultured cells, Mancias et al. have shown that ferritinophagy via selective cargo receptor NCOA4 regulates erythropoiesis [49]. Recently, a mice study has suggested that NCOA4-mediated ferritinophagy in macrophages plays essential roles to maintain erythropoiesis [50]. There is another form of selective autophagy termed crinophagy, whereas the secretory granules are directly fused with late endosomes or lysosomes for disposal [51]. As blood cell differentiation involves the secretion of granules or vesicles that may cause local tissue damage, crinophagy may play an important role to limit such damage through the uptake of granules by barrier cells and regulatory stromal cells while maintaining blood cell homeostasis.

In selective macroautophagy, the shape and size of the phagophore are regulated by the cargo itself and a variety of adaptor proteins including p62, optineurin (OPTN), NDP52, and NBR1 to link cargoes to the autophagy machinery [52]. Most of these adaptor proteins are associated with hematopoietic cell maintenance and blood malignancies such as acute myeloid leukemia (AML). AML is a heterogeneous disease and a common type of acute leukemia with poor survival and prognosis. It results from aberrant changes in the hematopoietic cells, which block myeloid differentiation and suppress hematopoiesis [53]. Selective autophagy receptor p62 is essential for hematopoietic cell growth and mitophagy to maintain mitochondrial integrity. Nguyen et al. have suggested that by depleting p62, mice develop myeloid leukemia and exhibit impaired mitophagy [54]. In hematological malignancies, OPTN has been identified as a prognostic biomarker for AML development and progression [55]. Moreover, simultaneous inhibition of autophagy receptors including p62, OPTN, and NDP52 sensitizes AML cells to the chemotherapy drug cytarabine [56]. In AML patients, quantitative real-time PCR analysis has suggested the downregulation of selective autophagy marker NBR1 expression along with other autophagy proteins such as LC3, Beclin1, UVRAG, and Rubicon [57].

Microautophagy can be either selective or nonselective. Nonselective microautophagy (generally refers to as microautophagy) is the random engulfment of intracellular organelles by tubular invagination or protrusions of the lysosomal membrane and the degradation of those bulk cytosolic cargos [58]. This kind of microautophagy is frequently spotted in mammalian cells. Conversely, selective microautophagy involves the degradation of substrates including lipid droplets (microlipophagy), peroxisomes (micropexophagy), and portions of the nucleus in a manner called piecemeal microautophagy of the nucleus (PMN) [59]. These forms of selective microautophagy are generally observed in yeasts. However, the molecular and functional details of microautophagy in hematopoietic cells and leukemia remain unknown.

Cellular proteins with a KFERQ-like motif are selectively degraded by lysosomes through CMA. During this process, KFERQ-like motif-bearing proteins are recognized and bound by a cytosolic chaperone named heat shock protein family A (Hsp70) member 8 (HSPA8) to form a translocation complex (Figure 1). Then, the complex is delivered to the lysosomal membrane where LAMP2A multimerization allows the channel formation to deliver proteins into the lysosomal lumen by lumenal chaperone heat shock protein 90 (HSP90). Lysosomal hydrolases break down the protein and release amino acids that are transported into the cytosol [60]. CMA is critical for HSC homeostasis. It has been suggested that quiescent HSCs derived from young mice have higher basal CMA activity than those derived from older mice [61]. In contrast, the deletion of Lamp2a blocks CMA, which indicates that CMA is vital for preserving HSC functionality and prevents the depletion of activated HSCs. Moreover, cells lacking CMA due to Lamp2a ablation produce less ATP, reduce glycolytic and oxidative phosphorylation activity for quiescent HSCs, generate more ROS, and accumulated proteins involve in metabolism. Myelodysplastic syndrome (MDS) AML patients exhibit resistance to azacytidine and poor survival as a result of a significant defect in CMA caused by a lack of LAMP2 expression, and lysosomal autophagy inhibitors such as CQ can be effective on AML cells lacking LAMP2 [62]. CMA activation in hematopoietic malignancies is also associated with the elimination of oncogenes such as mutated TP53 (tumor protein 53) and MLLT11 (myeloid/lymphoid or mixed-lineage leukemia translocated to 1q) [63, 64]. Similarly, AML patients with low LAMP2 expression levels have poor overall survival due to decreased CMA activity [65]. In chronic lymphocytic leukemia (CLL), AKT inhibitors and mTOR inhibitors (Torin-1) effectively restore CMA activity while removing prooncogenic proteins involved in leukemia initiation and progression [66]. Altogether, it appears that increasing CMA activity in the treatment of hematological malignancies will be an excellent therapeutic strategy, either alone or when combined with conventional chemotherapy.

While autophagy acts as a quality control process, the cargo selection process could be independent of autophagy. Noncanonical autophagy is a good example that utilizes some components of the macroautophagy machinery, while cargo identification and sequestration for autophagy occur upstream of autophagosome formation to process the extracellular cargo for lysosomal degradation [67, 68]. For instance, in LC3-associated phagocytosis (LAP), LC3 binds to phagosomes, modulating immune responses, antigen presentation, LC3-associated endocytosis, and tumor immune tolerance [69–72]. LAP functions in phagosome maturation and subsequent signaling events, while it is associated with the incorporation of the most upstream autophagic players such as *Beclin1*, *VPS34*, and *VPS15* [73]. Importantly, LAP catalyzes the formation of a single membrane LAPosome other than double-membraned autophagosomes, whereas LAP exclusively utilizes the ultraviolet radiation resistance-associated gene (UVRAG) which contains the class III phosphatidylinositol 3-kinase (PI3K) complex (Figure 1, lower panel) [74]. Moreover, Rubicon (RUN domain protein as Beclin1-interacting and cysteine-rich-containing) has been identified as a key modulator of LAP that is noncanonical and independent of ULK kinases [75]. It is a negative regulatory protein of the class III PI3K complex in macroautophagy [76] and is critical for the localization of phosphatidylinositol 3-phosphate (PI(3)P) and stabilization of the active NOX2 (NADPH oxidase 2) complex to promote optimal ROS generation during successful LAP [74]. Several noncanonical autophagy has been previously identified, such as ammonia-induced ULK1/2-independent autophagy where it is suggested that ammonia-induced autophagy is independent of ULK1/2 kinases but requires *ATG5* [77]. Recently, acetyltransferase p300-dependent class III phosphoinositide 3-kinase VPS34 activation and deacetylation have been demonstrated as noncanonical autophagy in which the upstream kinases of VPS34 such as ULK1 and AMPK can be bypassed [78]. This alternative autophagy underscores the involvement of canonical autophagosome biogenesis and the possibility of autophagy regulation in pathological consequences.

LC3-associated phagocytosis suppresses AML progression in bone marrow macrophages. The increased phagocytic activity of LAP led to the activation of the stimulator of the IFN genes (STING) pathway, which ultimately suppresses AML cell growth [79]. Furthermore, LAP promotes myeloid cell immunity to tumors, while single-cell RNA sequencing in tumor-associated macrophages (TAM) suggests that LAP defects trigger proinflammatory gene expression and type I interferon responses through STING modulation [72].

### 3.1. Macroautophagy Mechanism and Its Roles in Hematopoiesis

Autophagy induction is triggered through distinct signaling cascades under starved conditions and pathogen infection phage that results in the repression of the mammalian target of rapamycin (mTOR) [80, 81]. Opposingly, in nutrient-rich conditions, mTOR switches on and prevents ULK1 activation and disrupts the ULK1 association with adenosine monophosphate activated protein kinase (AMPK) [82]. During low glucose content, AMPK is activated and mTORC1 is inhibited, allowing ULK1 phosphorylation by AMPK interaction and eventually activating autophagy [82]. Next, ULK1 forms a tetrameric complex with FAK family kinase-interacting protein of 200 kDa (FIP200), ATG13, and ATG101 to recruit the VPS34 complex for phagophore isolation and autophagosome initiation [83, 84] (Figure 2). The class III PI3K catalytic subunit VPS34 then interacts with ATG14, VPS15, and Beclin1 to form a protein complex (PI3KC3) which is essential for the initiation and expansion of autophagosomes [85]. Furthermore, PI3KC3 synthesizes the lipid phosphatidylinositol 3-phosphate (PI(3)P), which recruited WD-repeat protein interacting with phosphoinositide (WIPI) proteins, and subsequently, WIPIs recruit Atg16L1 that conjugates with the autophagosome marker microtubule-associated protein 1A/1B-light chain 3 (LC3; mammalian ortholog of ATG8) through the Atg5/12/16L1 complex recruitment [86]. During autophagosome maturation, LC3 translocates from the cytosol to the isolation membrane where the cysteine protease ATG4 cleaves pro-LC3 to generate LC3-I. Then, LC3-I is subsequently transferred by ATG7 to the expanded phagophore membrane where LC3-I travels through Atg3, lipidated to LC3-II, and attached to the autophagosomal membrane [87]. In parallel, the ATG5/12/16L complex stimulates the conjugation of LC3-I to phosphatidylethanolamine (PE) to form lipidated LC3 (LC3-II) which binds to receptor molecule p62 inside the inner and outer membranes of the autophagosome [88] (Figure 2). Additionally, LC3-II is also involved in phagophore extension and closure during autophagosome formation. The next step is the fusion of the matured autophagosome with the hydrolase enzyme containing lysosome, referred to as autophagolysosome [89].

Hematopoietic cells are relatively low in basal autophagy, particularly in abundant nutrients and energy supply. The basal level of autophagy contributes to cellular homeostasis and protects cells against detrimental and dysfunctional organelles. Under nutrient-deprived conditions, normal blood cells undergo autophagy, which provides energy and building blocks for cellular homeostasis by breaking down misfolded proteins and damaged organelles [90]. During normal hematopoiesis, autophagy clears mitochondria, keeps a lower ROS level, and maintains the HSC stemness and genomic stability. Conversely, in malignant hematopoiesis, autophagy leads to excessive cell survival, low glucose consumption, and cell proliferation, which in turn induces malignant invasion by promoting the differentiation of leukemic cells. Besides, impaired autophagy during hematopoiesis may result in damaged organelles, protein aggregation, and excessive ROS and mitochondrial mass accumulation, leading to DNA damage and genomic mutation in the hematopoietic system (Figure 2) [91, 92]. Consequently, autophagy deficiency may cause HSC impairment, aberrant myeloproliferation, and severe anemia, while malignant blood cells, such as AML cells, exhibit a higher proliferation rate, apoptosis, and drug (chemotherapy and radiotherapy) resistance [93]. Interestingly, studies have also linked macroautophagy to AML development. In AML patients, high expression levels of key autophagic genes such as *ATG7* and *Beclin1* are associated with poor clinical outcomes and short remission duration [94, 95].

In contrast, genetic inhibition of murine *Atg5* and *Atg7* can prolong leukemia survival and delay the elimination of leukemia-initiating cells [96]. Altogether, these data indicated that both heightened and deficient autophagy activity may require for the malignant progression in AML. Currently, for most patients suffering from AML, primary and secondary drug resistance continues to be a major problem. Therefore, researchers are primarily exploring the mechanisms of drug resistance to develop next-generation AML therapies and designing combination regimens with the ultimate goal of eliminating all subclones of the disease and increasing the cure rate for AML. Overall, the canonical autophagy-dependent and canonical autophagy-independent (noncanonical) functions of the *ATG*s have been summarized in Table 1.

### 3.2. Autophagy in the Maintenance and Survival of HSCs

Autophagy is crucial to the proper development, survival, and maintenance of HSCs during acute metabolic stress. HSCs lacking autophagy exhibit impaired self-renewal activity as well as other characteristics similar to those of aging HSCs, which indicates the essential role of autophagy in regulating HSC pluripotency and preventing HSC aging (Table 2). Mice with HSC-specific conditional deletion of *Atg12* result in defective HSC self-renewal and metabolism, myeloid cell expansion, and premature blood aging [36]. Furthermore, MitoTracker Green (MTG) labeling, immunofluorescence staining for TOM20, and elevated levels of p62 have suggested an increased mitochondrial mass and depleted bulk autophagy in *Atg12*-deficient HSCs. This suggests that PINK1-dependent mitophagy is activated in HSCs. Conditional deletion of *Atg7* in adult mice HSCs leads to impaired HSC activity, enhanced mitochondrial metabolism, and severe myeloproliferation, which indicates that *Atg7-*deficient bone marrow cells are only capable of sustaining short-term hematopoiesis [165, 166]. However, *Atg7-*deficient neonatal HSCs have minimal effect on hematopoiesis or metabolic state, as they display long-term regenerative capabilities similar to wild-type neonatal HSCs [166]. Similarly, mice with HSC-specific ablation of *Fip200* and *Atg5* have shown significant reduction in HSCs, increased ROS and HSC proliferation, decreased HSC reconstitution ability in the bone marrow, and survival defects including severe anemia and lymphopenia [167, 168]. Mechanistically, it appears that impaired autophagy-mediated mitochondrial clearance underlies the functional defects in HSCs. In addition to maintaining normal hematopoiesis, macroautophagy plays an important role in regulating leukemic cell survival and the progression of AML [169]. It has been demonstrated that the inactivation of ATG7 or ATG5 in mice increases survival and suppresses functional leukemic HSCs as these are the principal cell types in AML development [96]. Similar to the murine AML model, AML patients have also shown increased Notch signaling and lower levels of autophagy in their HSCs [170]. Since inhibiting the Akt-mTOR pathway can trigger autophagy activation and control HSC homeostasis, thus this network is essential for self-renewal, survival, differentiation, and preventing HSCs from becoming leukemia stem cells (LSCs) [171]. Collectively, these findings have demonstrated the indispensable role of autophagy in HSC biology.

### 3.3. Autophagy in the Maintenance of Hematopoietic Progenitor Cells

**Figure 3:**
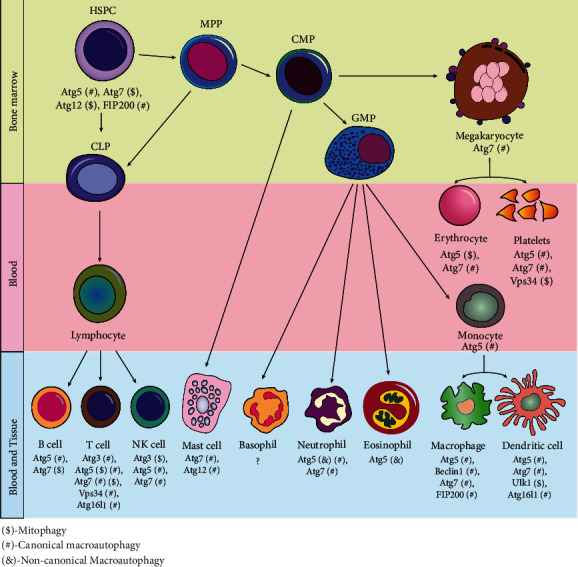
Autophagy-related genes involved in hematopoietic differentiation. Schematic representation indicated that different autophagy and marker proteins play vital roles during hematopoiesis. Specific stages of hematopoietic cell differentiation require the putative mechanistic involvement of different autophagy genes as well as multiple autophagy factors. HSPC: hematopoietic stem and progenitor cell; CMP: common myeloid progenitor; CLP: common lymphoid progenitor; GMP: granulocyte-macrophage progenitor; NK: natural killer cell; PP: multipotent progenitor.

Autophagy plays an important role in the maintenance of hematopoietic progenitors in the bone marrow (BM) (Figure 3). Mice with *Atg7* deficiency in the HSPC compartment result in significantly reduced common myeloid progenitors (CMPs) and common lymphoid progenitors (CLPs), increased mitochondrial ROS, DNA damage, apoptosis, and consequently induced severe myeloid proliferation leading to AML [172]. Further, histological analyses have suggested that *Atg7*-deficient mice develop myeloid leukemia and die as a result of BM failure. Together, this study has demonstrated that autophagy plays a crucial role in maintaining adult HSPCs and protection against leukemia development. Using flow cytometry-based autophagic vesicle quantification, as well as measuring LC3-II and p62 levels, it has been reported that human HSPCs have a higher basal autophagic flux than more differentiated myeloid or erythroid progenitor cells [173]. Since autophagy suppression via *ATG5* or *ATG7* knockdown results in decreased HSPC frequencies, higher autophagy flux is essential for the maintenance of myeloid and erythroid progenitor cell function [173]. However, such a decrease in HSPCs occurs not as a result of impaired differentiation but rather due to reduced cell cycle progression and increased apoptosis. Another study has suggested that the loss of *Atg5* increases the mitochondrial oxidative stress in neonatal HSPCs [92]. Even though p62 accumulates in immature bone marrow cells of *Atg5*-deficient mice, p62 deletion does not restore defective HSC functions, which indicates that p62 is not necessary for *Atg5*-dependent hematopoietic regulation [92]. Autophagy inhibition by mTOR signaling modulates myeloid progenitor-derived macrophage differentiation [174]. Together, these data have shown the importance of autophagy in reducing cellular stress, promoting survival, and regulating HSPCs.

### 3.4. Autophagy in Lymphopoiesis

Autophagy renders a pivotal role in lymphoid differentiation and maturation. Mechanistic insights using experimental mouse models suggest that the deletion of *Atg5* or *Atg7* inside the T-cell and B-cell compartments has perturbed the fundamental autophagy process and dysregulated the cellular renewal, differentiation, and immune cell functions during lymphoid maturation (Figure 3).

#### 3.4.1. Autophagy in B Lymphocyte Development

During early embryogenesis, the development of B lymphocytes initiates in a stepwise manner such as pro-B cells, pre-B cells, and immature B cells from HSCs inside the bone marrow [175–178]. Afterward, immature B cells migrate and secrete antibodies to the secondary lymphoid organs where they get fully matured. B lymphocytes are categorized into B-1 and B-2 lymphocytes depending on their cell surface marker expression properties [179]. Matured B cells further differentiate into either quiescent memory B cells or long-lived antibody-secreting plasma cells [180]. As a result of apoptosis, autophagy-defective plasma cells and memory B cells are unable to synthesize proteins continuously and eventually come across misfolded protein aggregation [181, 182]. The first evidence concerning the necessity of autophagy during B-cell development comes from the conditional knockout of the *Atg5* mice model, whereas B lymphocyte-specific *Atg5* ablation results in defective B-cell development during pro-B-cell to pre-B-cell transition stages with a substantial decrease in B-1 lymphocytes inside the bone marrow [183]. B-1 lymphocytes are more likely to undergo cell death, which affects their numbers more than unaffected peripheral B-2 cells. This indicates that autophagy may be a critical process in peripheral B-1 cells. Conditional deletion of *Atg5* in B cells caused endoplasmic reticulum stress and impaired antibody responses inside the plasma cells, impairing plasma cell homeostasis and long-term humoral immunity [184–186]. Furthermore, *Atg5* autophagy-deficient mice develop anemia and extramedullary hematopoiesis (EMH), most likely due to the absence of proinflammatory cytokines [187].

#### 3.4.2. Autophagy in the Quiescence and Development of T Lymphocytes

Autophagy plays an important role in T lymphocyte homeostasis. Deficiency of *Atg5* in mice embryonic stem cells shows full T lymphocyte maturation, but the peripheral T and B lymphocytes and total thymocytes are reduced [188]. Targeted deletion of *Vps34* and *Atg16l1* in the T-cell compartment of aged mice models impairs the normal development of innate natural killer (NK) T lymphocytes [189, 190]. Under normal circumstances, invariant natural killer T (iNKT) cells display upregulated mitophagy during thymus development. However, *Atg7*-ablated mice within the T-cell compartment perturb cellular differentiation of iNKT cells including the enhanced susceptibility of iNKT cells to apoptosis and the accumulation of mitochondrial superoxide species [191], impaired peripheral survival of memory CD8^+^ T cells, and subsequently block mature iNKT cell formation in the peripheral lymphoid organs [192]. Such autophagy deficits result in the accumulation of intracellular mitochondrial ROS and apoptosis. Similarly, mice with a T lymphocyte-specific deletion of *Atg5* or *Atg7* indicate a cell-autonomous profound decrease in the iNKT cell population, increased ROS and mitochondrial content, and increased apoptosis and survival defects in T cells [193, 194]. Together, these findings highlight the unique roles of *Atg5* and *Atg7* for the quiescence and development of T lymphocytes. Similar scenarios are also observed in mice with *Atg3*-deficient T lymphocytes [195]. Furthermore, T-cell-specific deletion *Atg5* or *Atg7* results in autophagy deficiency and defective T lymphocyte production [196]. Moreover, conditional ablation of *Atg5* or *Atg7* in peripheral blood-derived CD8^+^ T cells (also known as cytotoxic T lymphocytes) induces defective autophagy flux, pathogen infection, and survival defects in memory T cells [197]. Inducible knockout of *Atg5* in mouse CD8^+^ T cells results in concomitant p53 activation, higher ROS, apoptosis, hypoxia in lymphoid tissues, and increased susceptibility to viral infection [198]. Autophagy is also essential for cell transplantation studies, as *Atg16l1* deficiency leads to an exacerbated graft-versus-host disease (GVHD) in a mouse model of allogeneic HSC transplantation (allo-HSCT), whereas *Atg16l1*-deficient allo-HSCT recipients with GVHD displayed increased T-cell proliferation due to increased dendritic cell (DC) numbers [199]. Collectively, these results demonstrate that T lymphocytes require autophagy to suppress cell death and maintain survival in response to virus and pathogen infection.

#### 3.4.3. Autophagy in Natural Killer Cell Development, Maturation, and Function

Natural killer (NK) cells are specialized large lymphocytes that play critical roles in recognizing and clearing virally infected and targeted tumor cells while ensuring innate and adaptive immune responses against viral infections and pathogen attacks [215]. NK cells are derived from CLPs, while their development takes place in the bone marrow, and differentiated into immature or innate NK cells (iNKs) and eventually into mature NKs (mNKs) [215–218]. In addition to their well-established roles in antitumor and antiviral immune response, NK cells are also responsible for the production of proinflammatory cytokines and chemokines, such as interferon gamma (IFN-*γ*), tumor necrosis factor (TNF), chemokine (C-C motif) ligand 3 (CCL3), CCL4, and CCL5, regulating tissue and immune homeostasis [219].

Autophagy plays a crucial role in regulating NK cell survival, development, and responses against infections. Mouse bone marrow-derived iNK cells show basal autophagy activity including LC3 lipidation and p62 degradation that may be attributed to the highly proliferative murine mNK cells. It has been reported that canonical autophagy component *Atg5* is essential for the proper development of mNK cells, while mice with *Atg5* deficiency displayed high viral titers due to a drastically reduced number of peripheral mNK cells [220]. Wang et al. suggested that mice with NK cell-specific ablation of *Atg5* showed severe reduction in iNKs and mNKs within the bone marrow and spleen, leading to the accumulation of ROS and damaged mitochondria [205]. They also indicated that *Atg7* silencing impairs iNK cell autophagy and functional NK cell development in mice. Therefore, autophagy may have a role to play in different stages of NK cell maturation and proliferation during homeostasis.

Autophagy may not be required during activated NK cell proliferation, but it is critical during the transition from effector to long-lived memory cells, particularly when the autophagosome machinery component *Atg3* is absent [204]. Moreover, autophagy modulator mTOR (mechanistic target of rapamycin), a serine/threonine kinase, also plays an essential role in NK cell development. Studies also suggested that mTOR and its related complexes including RPTOR (regulatory associated protein of mTOR complex 1) and RICTOR (RPTOR independent companion of mTOR complex 2) participate in NK cell development. When RPTOR or RICTOR is depleted, two distinct mTOR complexes (TORC1 and MTORC2) are destabilized, while rapamycin (mTOR inhibitor) administration impairs NKP differentiation without affecting autophagy [205]. This implies that mTOR participates in NK cell development but may be independent of autophagy. Nevertheless, these findings indicated that autophagic responses may influence NK cell biology.

### 3.5. Autophagy in Myelopoiesis and Granulopoiesis

Myelopoiesis is a stepwise differentiation and maturation of HSCs to CMPs by terminal differentiation which led to the production of monocytes and granulocytes including neutrophils, basophils, and eosinophils. This process is subdivided into monocytopoiesis and granulopoiesis [221]. However, it remains elusive how autophagy mediates monocyte and granulocyte differentiation.

#### 3.5.1. Autophagy in Neutrophil Differentiation and Degranulation

Granulopoiesis is the sequential differentiation of GMPs to become eosinophils, neutrophils, basophils, and macrophages inside the bone marrow [221]. Granulocytes, also known as polymorphonuclear leukocytes (PMNL), are white blood cells, while neutrophil granulocytes are the short-lived and widely abundant cells of the host immune system, and their functional impairments lead to serious immunodeficiency syndromes [222]. There are at least four types of granules in neutrophils. Endoplasmic reticulum (ER) Golgi networks produce primary, secondary, and tertiary granules, while secretory vesicles are derived from endocytic origin. Neutrophil degranulation is an important process by which neutrophils kill the pathogen and modulate the immune response to an infection, while its inhibition can promote bacterial survival and pathogen infection. It has been shown that not only phagocytosis kills microbes but extracellular traps (ET) named neutrophil extracellular traps (NETs) for neutrophils also kill microbes in the extracellular space. NETs are made up of extracellular fibers, granular proteins, and deoxyribonucleic acid (DNA), while NET formation serves as a defense against infection by the innate immune system [223]. Therefore, neutrophil plays important roles in the body's innate immune defense via migrating to the inflammation site and controlling microbial infection by phagocytosis, degranulation, forming NETs, and secreting antimicrobial compounds. However, there is no conclusive evidence that autophagy is involved in the NET formation. Germic et al. indicated that the formation of extracellular traps by eosinophils and neutrophils is not dependent on *Atg5*-regulated autophagy, as both murine *Atg5* knockout eosinophils and neutrophils exhibit normal extracellular trap (ET) formation [224]. Using human and mouse neutrophils, their further investigation has demonstrated that NET formation was significantly blocked after pretreatment with class III PI3K inhibitors such as 3-methyladenine (3-MA) and wortmannin. Conversely, bafilomycin A1 and chloroquine (CQ), so-called late autophagy inhibitors, have no effect on NET formation, which suggest the autophagy-independent role of *Atg5* in NET formation [224]. Taken together, the picture remains unclear whether autophagy is indeed involved in the NET formation.

During neutrophil granulopoiesis and degranulation, autophagy plays an important role in neutrophil granule secretion and its degranulation (Figure 3) [225]. For instance, *Atg5*-deficient mice have shown minimal degranulation from primary and secondary granules. To test whether *Atg5* plays an autophagy-independent role in neutrophil degranulation, Bhattacharya et al. generated the *Atg5*-deficient mice model from myeloid cells that resulted in reduced degranulation of primary, secondary, and tertiary granules from bone marrow neutrophils (BMNs) [225]. Although myeloid-specific autophagy deficiency leads to increased circulating neutrophil numbers and subsequent recruitment to inflammation sites, these neutrophils were unable to effectively mediate inflammation due to their reduced effector functions. As with many biological phenomena, there is still much to learn about the consequences of suppressed autophagy on neutrophil degranulation. *Atg5* deletion from mice myeloid cells accelerated the neutrophil differentiation process and the proliferation of neutrophil precursor cells inside the bone marrow, which eventually leads to an accumulation of neutrophils in the bone marrow, blood, spleen, and lymph nodes [226]. Furthermore, *Atg7-*deficient mice exhibit impaired mitochondrial respiration, excessive glycolysis, reduced ATP production, and defective lipophagy during neutrophil differentiation. Nevertheless, the exact pathway by which lipophagy is directed into the autophagic machinery requires further investigation.

#### 3.5.2. Autophagy in Eosinophil Biogenesis

Similar to neutrophils, eosinophils and basophils are also granulocytes that arise from the bone marrow but occur in relatively low numbers in human peripheral blood. A response to inflammation triggers the maturation of eosinophils in the bone marrow and then leads to its migration and activation in inflamed tissues. In contrast to neutrophils, it remains largely unknown how autophagy regulates eosinophil hematopoiesis and functions because eosinophils are rare and poorly studied. During an innate immune response, no defects in the formation of eosinophil extracellular traps (EETs) were observed in *Atg5* knockout mouse eosinophils or autophagy-deficient human eosinophils (Figure 3) [224]. Rapamycin, however, effectively inhibits eosinophil differentiation by blocking mTORC1 and significantly reduces eosinophil counts in the peripheral blood and bone marrow [227]. Surprisingly, an increased number of eosinophil lineage-committed progenitors were observed in the bone marrow after rapamycin inhibited eosinophil differentiation. The same group also found an increase in eosinophil lineage-committed progenitors upon the murine myeloid cell-specific deletion of mTOR [228].

However, their LysM-cre mice strain is questionable because this group used that mice strain to suppress mTOR expression in eosinophils, which is normally used to determine neutrophilic, macrophagic, and dendritic cell populations, but not eosinophils. Collectively, these two studies indicated that mTOR modulates eosinophil differentiation in different ways, possibly because mTORC1 and mTORC2 may have distinct functions. So far, autophagy has not been reported to be involved in basophil hematopoiesis.

#### 3.5.3. Autophagy in Mast Cell Function and Degranulation

Mast cells (MCs) are granule-containing immune cells that are found in nearly all vascularized tissues and contribute to the generation of protective innate immunity against infection. Furthermore, activated MCs regulate adaptive immune responses by influencing lymph node composition [229]. Despite this, very little is known about how autophagy contributes to MC function. It has been reported that under adequate nutrient supply, bone marrow-derived mast cells (BMMCs) exhibit autophagic flux, and LC3-II is found in their secretory granules [230]. When *Atg7* is deleted from BMMCs, degranulation has been severely impaired, but cytokine production is not affected, suggesting that autophagy may not be essential for mast cell differentiation but critical for their degranulation.

#### 3.5.4. Autophagy in Monocyte and Macrophage Differentiation

Monocytes are bone marrow-derived white blood cells or leukocytes, circulating inside the blood, and upon migrating from the bloodstream into tissues, monocytes are terminally differentiated into macrophages and dendritic cells [231]. Such immune cells are involved in various cytokine induction and antigen presentation while conferring innate immunity and tissue homeostasis [232], while macrophages are multifaceted innate immune phagocytes that serve as a first-line host defense against intracellular pathogen invasion by mounting proinflammatory responses via phagocytosis, releasing cytokines and renovating damaged tissues [233]. A study reported that colony-stimulating factor-1 (CSF-1) or granulocyte/macrophage colony-stimulating factor (GM-CSF) induces autophagy and is essential for proper macrophage differentiation in human and murine monocytes [234]. Moreover, the differentiation signal prevents ATG5 cleavage and activates c-Jun N-terminal kinase (JNK) to release Beclin1 from BCL-2, both of which are essential for the initiation of autophagy. Since the Beclin1 knockdown and autophagy inhibitors including 3-MA and CQ cause apoptosis via caspase activation and prevent differentiation and cytokine production, therefore autophagy is critical in transitioning from monocyte apoptosis to differentiation (Figure 3). In 2020, Wang et al. reported that mice with myeloid cell-specific deletion of *beclin1* and *Fip200* induce defective lymphoid and myeloid cell homeostasis and impaired macrophage differentiation [206]. Conversely, excision of other core autophagy genes including *Atg5*, *Atg7*, *Atg14*, and *Atg16l1* from the mice macrophages showed moderate to severe level vulnerability to pathogen infection and bacterial burden. To determine the autophagic activity of all these genes, degradation of the p62 protein and lipidation of LC3 are measured. Collectively, it appears that certain autophagy genes, but not all, have a key role in maintaining immunity quiescence within tissue-resident macrophages. Circulating monocytes derived from CMPs can generate macrophages and dendritic cells inside tissues through cellular differentiation [235]. However, detailed mechanistic insights and molecular events have not been elucidated. In contrast, mice monocytes and macrophages lacking *Atg5* are more likely to be infected by intracellular pathogens, suggesting that *Atg5* expression in such phagocytic cells supports intracellular pathogen resistance [236]. However, in this study, classical autophagosomes were missing during lysosomal fusion with phagosomes and *Atg5*-dependent intracellular killing of pathogens, which suggests a classical autophagy-independent role of *Atg5* in intracellular membrane dynamics. Similarly, myeloid lineage-specific *Atg7*-deficient mice's macrophages showed increased bacterial uptake with accumulating p62 level when infected with *Mycobacterium tuberculosis* [237]. It is presumable that autophagy plays similar roles in monocytes/macrophages and granulocytes of hematopoietic origin, although the direct mechanistic insights of autophagy in blood monocytes and macrophages remain poorly understood.

#### 3.5.5. Autophagy in Dendritic Cell Immune Response

Dendritic cells (DCs) are a special kind of antigen-presenting cells, derived from HSCs, which sequentially differentiate into CMPs and CLPs. DCs from the CMPs capture antigens in peripheral tissue and then migrate to the lymphoid organs for immunity. On the other hand, DCs arise from the CLP origin and are found in the lymph node T cells and thymic medulla to initiate immune tolerance and T-cell immunity [238]. As previously mentioned, autophagy machinery is utilized to deliver the pathogenic organelles into autophagosomes and its subsequent fusion with lysosomes, it is believed that cargos from lysosomal degradation are delivered to T helper cells by major histocompatibility complex (MHC) class II molecule to mediate the adaptive immune response [239]. Lee et al. have suggested that mice with *Atg5* deficiency in DCs exhibited impaired CD4^+^ T-cell priming and rapid viral infection [240]. *Atg5*-deficient DCs also display a prominent defect in phagocytized antigen processing for MHC class-II molecule and phagosome-to-lysosome fusion. Previously, it has been stated that cytosolic receptors may be activated by pathogens, which then trigger autophagy. Therefore, *Atg16l1* autophagy is induced by the activation of bacterial sensors called nucleotide-binding oligomerization domain 1 (NOD1) and NOD2 [241]. Cooney et al. demonstrated that DCs from individuals with Crohn's disease expressing NOD2 or *Atg16l1* risk variants show abnormalities in autophagy induction, antigen presentation, and bacterial trafficking [242]. Autophagy activating kinase *Ulk1* plays a critical role in mitochondrial quality control in bone marrow-derived DCs (BMDCs). Mice lacking *Ulk1* in BMDCs have increased accumulation of damaged mitochondrial mass and cell death via caspase-1 activation [243]. Moreover, BMDC cytokine response is dependent on *Beclin1* autophagy. BMDCs infected with H1N1 influenza and lacking *Beclin1* are detected with lower levels of inflammatory cytokine response [244]. Thus, the ability to boost DC immunogenicity by inducing autophagy is a novel strategy for vaccination.

#### 3.5.6. Autophagy in the Maintenance of Megakaryopoiesis and Thrombopoiesis

Several human diseases are associated with a lack of autophagy, while their role in platelet function has only recently been explored. Thrombocytes or platelets are colorless, small, and flowing cell fragments in the bloodstream, generated from the megakaryocyte progenitors (MPs), that immediately respond to blood vessel injury, form blood clotting, prevent bleeding, and assist in hemostasis [245]. It has been reported that resting human and mice platelets exhibit a wide range of autophagy proteins including ULK1, FIP200, ATG3, ATG5, Beclin1, ATG7, LC3-II, ATG12, ATG14, VPS15, VPS34, and UVRAG [246]. Autophagy is constitutively active in resting platelets, while platelet activation triggers autophagy and autophagy flux, as evidenced by agonist-induced loss of autophagy marker LC3-II [247]. Megakaryocyte and thrombocyte-specific ablation of *Atg7* indicated a markedly reduced LC3-II level, defective platelet aggregation, abrogated granule cargo packaging, impaired hemostasis, and thrombus formation. Similarly, hematopoietic cells lacking *Atg7* indicate an absence of LC3-II formation due to disrupted LC3 lipidation, which indicates the absence of autophagy. This leads to mitochondrial mass accumulation as detected by increased mitochondrial superoxide production and cell cycle dysfunction in *Atg7^−/−^* bone marrow Lin^−^ cells, which eventually disrupts megakaryopoiesis, megakaryocyte differentiation, and thrombopoiesis and results in aberrant platelet production, prolonged bleeding, and impeded hemostasis [248]. In another study, mice with megakaryocyte/platelet-lineage-specific ablation of *Atg5* contributed to impaired mitochondrial quality control and expanded mitochondrial mass inside the thrombocytes and blocked mitophagy [249]. Using VPS34- (vacuolar protein sorting 34-) deficient mice, Liu et al. further indicated that VPS34^−/−^ mice exhibited impaired mTOR signaling and arterial thrombosis, significantly reduced thrombus formation, and altered basal level of autophagy in resting platelets [213]. Collectively, these data suggested that platelet autophagy, especially platelet mitophagy, is crucial for hemostasis and thrombus formation.

Autophagy has also been studied in invertebrate hematopoiesis to a lesser extent. *Drosophila*'s hematopoietic system is an excellent genetic model for studying invertebrate hematopoiesis. It has been reported that homozygous *Atg6* loss causes melanotic blood cell masses in *Drosophila*, whereas ubiquitous expression of *Atg6* completely rescues the phenotype of blood cell tumors [250]. Melanotic blood cell masses are also observed in *Atg7* and *Atg13* mutated *Drosophila*. Immunohistochemical analyses using the GFP antibody labeling have suggested that homozygous *Atg6* mutant larvae have blood-like melanotic masses that confirm that hematopoietic cells are the source of these masses.

## 4. Expression Profile of ATG Proteins in Hematopoietic Cells and AMLs

**Figure 4:**
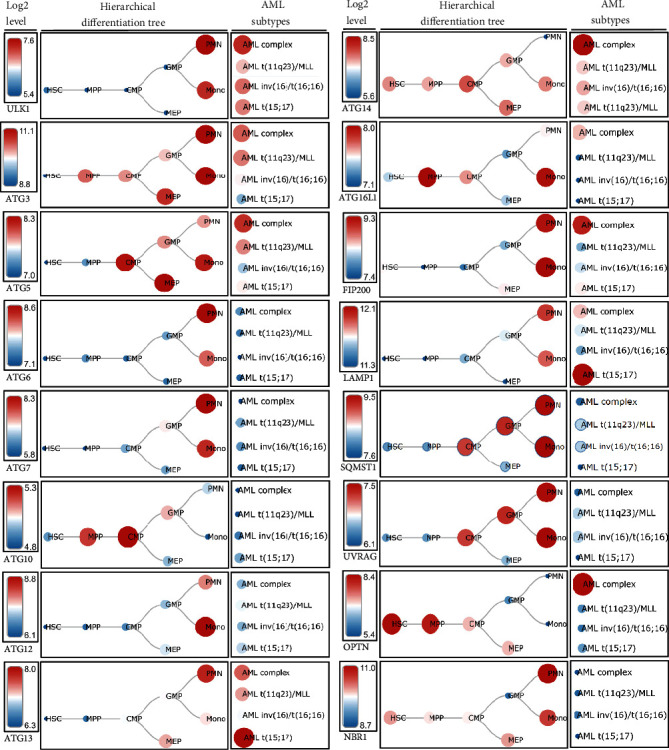
Expression of different autophagy markers in hematopoietic cells and AMLs. Schematic representation indicated the expression level of different *ATG* genes, autophagy-related factors, and different ATG receptors in both hematopoietic cells and various types of acute myeloid leukemia. PMN: polymorphonuclear cells; MPP: multipotential progenitors; MLL: mixed-lineage leukemia; AML t(15;17): AML with t(15;17); AML inv(16)/t(16;16): AML with inv(16)/t(16;16); AML t(11q23)/MLL: AML with t(11q23)/MLL; AML complex: AML with complex aberrant karyotype. Data were generated from normal human samples and samples from the human AML cells using microarray-based gene expression profiling. The “BloodSpot” (https://servers.binf.ku.dk/bloodspot/) database was used to generate the hierarchical differentiation tree.

The catabolic nature of dynamic autophagy enables cells to maintain their self-renewal capacity, cellular homeostasis, and survival under stress conditions. In humans, ATG proteins are expressed differentially during normal hematopoiesis (Figure 4). Autophagy-activating kinase ULK1 and other core ATG proteins such as ATG6, ATG7, ATG13, autophagy factor FIP200, and autophagy receptor protein NBR1 are highly expressed in polymorphonuclear leukocytes and rarely expressed in MPPs, CMPs, MEPs, and GMPs. Conversely, core autophagy protein ATG5 is predominantly expressed in CMPs, MEPs, and monocytes. Autophagy receptor protein SQMST1 and tumor suppressor as well as an important macroautophagy regulator UVRAG are significantly expressed in PMNs, monocytes, GMPs, and CMPs. However, the basal levels of all autophagy proteins in the HSCs are relatively low.

The role of autophagy in various types of cancers has been extensively studied. However, the relationship between autophagy and hematopoietic malignancies remains controversial. Autophagy-related proteins such as ULK1, ATG3, ATG5, ATG13, and ATG14 were markedly expressed in various acute myeloid leukemias when comparing the microarray-based expression profiling of distinct ATG proteins from human AML samples with the human normal hematopoiesis. Nevertheless, the expression profiling of ATG proteins in AML samples is paradoxical. For instance, Jin et al. reported a lower expression of ULK1 in human AML cell lines [251], while Hwang et al. indicated a significantly higher level of ULK1 in FLT3-ITD-mutated AML cells compared to FLT3-wild-type AML cells, which ultimately suggested that inhibiting ULK1 in FLT3-ITD AML cells might be a promising therapeutic approach against FLT3-ITD-mutated AML [252]. Similar to the later study, it has been demonstrated that *Atg7* knockdown from patients' AML samples prolonged the overall survival after receiving chemotherapy [95]. As a result, increasing chemosensitivity by decreasing *Atg7* may be a potential strategy to improve treatment results for AML. Jin et al. also found significantly decreased ATG5 expression levels in AML samples. In contrast, a recent study found that an Egyptian cohort with newly diagnosed acute lymphoblastic leukemia (ALL) had considerably greater levels of ATG5 expression [253]. Moreover, Liu et al. suggested that *Atg5*-dependent autophagy may contribute to the development of murine AML induced by the MLL-AF9 fusion oncogene, resulting from the t(9;11)(p22;q23) translocation [254].

However, researchers suggested a Beclin1 autophagy-dependent but ATG5 autophagy-independent role in murine chronic myeloid leukemia (CML) model, whereas *Beclin1* knockdown, but not *Atg5* ablation, leads to a reduced leukemic burden and a significantly higher survival rate of targeted mice [255]. Collectively, autophagy plays dual roles depending on the kind of hematological malignancies. Nevertheless, more research is needed to clarify the relationship between autophagy and blood malignancies.

## 5. Current Challenges and Future Perspectives

Autophagy plays a critical role in the hematopoietic system by regulating the self-renewal of HSCs, differentiation, and development of lymphoid and myeloid progenitors and their precursor cells in response to cytokine signaling [256–258]. Several studies indicated that the deletion of ATGs such as FIP200, ATG5, and ATG7 decreases the number of HSCs and diminishes the reconstituting capacity of normal HSCs [167, 259]. Moreover, ATG7 or FIP200 deficiency in HSC results in abnormal myeloid expansion, accompanied by ROS accumulation and genomic instability, which contribute to the development of aggressive phenotypes in the hematopoietic system similar to anemia, lymphopenia, and splenomegaly. Besides, ubiquitous deletion of the core autophagy gene(s) from *in vivo* mice models leads to embryonically and neonatally lethal phenotypes [260–263]. To avoid such embryonic and neonatal lethality at the germline and to overcome the secondary effects associated with altered gene function in other tissues upon ubiquitous gene knockout, tissue-specific gene disruption strategies have been developed to study particular gene functions. Studying autophagy within a particular hematopoietic cell type or lineage-specific manner will allow us to determine the actual function of different *ATG*s in the hematopoietic system. However, such strategies might be difficult to apply in humans due to the molecular complexity of autophagy regulatory genes and ethical issues. Therefore, previous studies have been done using cell lines and animal model platforms. As an alternative approach, bone marrow transplantation strategies have been utilized to generate hematopoietic cell-specific knockout animals. To date, *Atg5*, *Atg7*, and *Beclin1* are the most studied autophagy genes concerning hematological physiology and pathobiology, while the other core autophagy genes such as *Ulk1*, *Atg3*, *Atg9*, *Atg10*, *Atg12*, *Atg13*, *Atg18*, and *Atg101* need further investigations. Moreover, direct mechanistic insights between impaired autophagy, ROS, and mitochondrial mass accumulation were partly lacking, possibly due to the nontransparent nature of the widely used *in vivo* mice model. To overcome the nontransparent nature of the mice models, the introduction of the *in vivo* zebrafish model having the optical transparency, high fertility, commercially available subcellular fluorescent dye, and ubiquitous and tissue-specific GFP : Lc3-expressing transgenic lines with time-lapse *in vivo* live-imaging technology has immensely alleviated the technical downfall and geared up the experimental outcomes to monitor dynamic autophagy in hematopoietic development, differentiation, and blood malignancies.

Autophagy is involved in the complex molecular signaling pathway; while numerous studies have demonstrated that autophagy promotes both cell survival and cell death, these studies have yet been unable to answer the basic question about autophagy's two functions. Moreover, autophagy is poorly understood at the molecular level and how tumor genetics, tissue type, and disease state affect its specific functions. Further research should be directed at understanding autophagy's role and status in cellular survival and death and clarify whether autophagy directly causes cell death and cell survival or whether such changes in the cell microenvironments are secondary to autophagy. Thus, it is also necessary to determine whether autophagy results from or causes changes in cellular metabolic processes. It is more likely that drug resistance may occur due to autophagy activation in leukemia cells during drug-induced apoptosis if autophagy acts as a secondary change.

Immune cells' activation and differentiation are also regulated by autophagy, while those immune cells' functions are regulated by their metabolism. Therefore, it would be beneficial to study tumor and host autophagy in immune cell metabolism to develop novel anti-AML therapies. We know that autophagy recycling is essential for the survival, metabolism, and proper functioning of AML cells, but does the same apply to other leukemias? Is autophagy in the host responsible for promoting leukemia cell growth, and if so, how does it work? How can the autophagy flux in human tissue be measured? Which steps should be targeted in the autophagy pathway for the development of inhibitors? Nevertheless, drugs that regulate autophagy are now being researched for the treatment of leukemia. A key feature of targeted therapy will be the selective induction of autophagy in leukemic cells. In such a scenario, a better starting point would be to investigate autophagy under hypoxic conditions. It is also challenging to determine which stages of AML blast formation require autophagy to protect the tumor cells and to clarify how autophagy functions during different stages of AMLs. Therefore, it is necessary to investigate specific pathways that activate autophagy in AMLs and other associated cellular processes including apoptosis and cell differentiation to identify targeted treatments and reverse AML cell growth. Recent advances have led to the development of more potent and specific lysosome inhibitors than chloroquine to develop a wide combination of different therapies, such as concanamycin A, which prevents the acidification of lysosomes and endosomes by inhibiting V-ATPase; pepstatin A, which inhibits cathepsins D and E; and E64d, which inhibits cathepsins B, H, and L [264]. However, autophagosome formation and cargo sequestration are not affected by the prevention of autophagosome degradation by lysosomal inhibitors. Mitophagy, for example, removes damaged mitochondria, preventing oxidative stress and the activation of apoptosis. Lysosomal inhibitors, nonetheless, may not be effective in reducing the rate at which mitochondria are sequestered by autophagosomes, and this could limit the drug's efficacy against AML effects that are reliant on mitophagy. Therefore, what level of the autophagy pathway would be optimal for inhibition remains an unanswered question. If the later steps, such as the lysosome, are inhibited, other metabolic scavenging pathways may also be blocked, which has also been demonstrated to be vital to tumor cell metabolism and growth. It is also possible for tumor cells to become toxic due to the aggregation of undelivered autophagosomes if the later stages of the autophagy pathway are targeted. Alternatively, inhibiting autophagy at the earlier stages, such as those phages involved in autophagosome biogenesis, would result in a buildup of damaged mitochondria and toxic protein aggregates, which would not be contained by the autophagosome anymore, resulting in constant exposure to these toxic insults to tumor cells. Nevertheless, a better outcome may be achieved in this context with drugs that block the initial phases of autophagy, such as ULK1 inhibitor (MRT68921) and VPS34 inhibitor (PIK-III or SAR405) [265–267]. Furthermore, it is important to determine whether specific autophagy pathways can be targeted or if targeting the general macroautophagy pathway will be sufficient. It may be possible to enhance therapeutic efficacy and minimize toxicity by targeting specific cargo adaptors in selective autophagy. Thus, developing more selective and potent autophagy/lysosome pathway inhibitors at different levels will facilitate preclinical validation studies. Collectively, a concrete knowledge and complete understanding of the complex autophagy network will help us to better develop more precise autophagy-modulating therapies and to evaluate which patients will be benefited from such treatments.

Basal autophagy could be monitored by isolating HSCs from the blood of AML patients. However, it is extremely challenging to study autophagy in living organisms, particularly in patients, despite methods for studying the autophagic flux in vitro. Therefore, further research is also needed to develop reliable and new methods to quantify autophagy flux in patient samples to adjust therapy and better target the autophagy pathway. Transcriptomic and proteomic analysis of patient cells could provide valuable insight into how treatment affects autophagy gene expression and how their expression might predict patient survival. Using RNA sequencing, an innovative approach to transcriptome analysis, it could be possible to predict whether or not these cells are undergoing autophagy activation. It is still possible to develop new therapeutic approaches in the future by investigating cargo receptors and selective autophagy. A growing body of evidence suggests that autophagy inhibition in normal tissues without driver oncogene mutations does not cause leukemia progression to fully invasive stages such as AML. As a consequence of these previous concerns, autophagy inhibition studies have been focused exclusively on AML; however, the new findings indicate that autophagy inhibition may be of critical importance for early-stage blood cancers, either as a prevention of metastases or as a prevention of leukemia from developing initially. It is believed that targeting either tumor or host autophagy is an important treatment strategy for AML because both are involved in the growth of the disease. While autophagy plays an important role in AML, there is still a need to fully understand its mechanisms across AML subtypes and treatment options.

## 6. Concluding Remarks

Autophagy has become an intriguing subject in hematopoiesis and related malignancies such as AML. Throughout this review, we have summarized the different forms of autophagy and discussed the hematopoietic-lineage-specific roles of different autophagy regulators. Since the blood progenitors and precursors derive from HSCs, targeting HSCs may be a better approach for treating AML. In addition, autophagy inhibitors might also be successfully used in clinical settings to treat AML patients by using newly developed drug delivery systems. However, introducing autophagy modulators in AML therapy should be considered in light of the potential toxicity they may cause in HSCs, even during the progression of malignant phenotypes. To overcome this potential toxicity, autophagy modulators need to be studied further.

## Figures and Tables

**Table 1:** Canonical autophagy-dependent and canonical autophagy-independent function(s) of different autophagy marker proteins.

Protein name	Autophagy step(s)	Canonical autophagy-dependent function(s)	Rf.	Canonical autophagy-independent function(s)	Rf.
ULK1 or ATG1	Initiation	Mitochondrial respiration, ATP production, lipid metabolism, and mitochondrial and ribosomal clearance	[97, 98]	Cell death and apoptosis, endocytosis, immune signaling, antiproliferative and antineoplastic effects in MPNs, ER-to-Golgi trafficking, cellular homeostasis, ammonia-induced autophagy, and endosomal trafficking	[99–106]
ATG2A or ATG2B	Elongation	Regulates lipid homeostasis, promotes Atg9-Atg18 interaction, and programmed cell size reduction	[107–109]	iDISC-dependent caspase-8 activation, apoptosis, and lipid droplet localization	[110, 111]
ATG3	Elongation	Induces HIV infection and cell death	[112]	LAP, endosomal trafficking, and apoptosis	[74, 113, 114]
ATG4B or ATG4D	Elongation	Sense balance and otoconial development induce HIV infection and cell death	[112, 115]	LAP, mitochondrial dysfunction, and apoptosis	[74, 116]
ATG5	Elongation	Maintenance of innate lymphocytes, skeletal homeostasis, and antiviral immune responses	[117–119]	Immunity, intracellular pathogen killing, apoptosis, and adipogenesis	[120–123]
ATG6 or Beclin1	Nucleation	Induces HIV infection and cell death	[112]	Apoptosis, cell death, cancer cell growth, embryogenesis, tumor suppression, STAT3 phosphorylation, DNA damage repair, receptor degradation, and cytokinesis and induces viral transmission and improves the life span	[124–132]
ATG7	Elongation	Maintains cellular and behavioral responses and regulates potassium (K^+^) level in hypokalemia	[133, 134]	Cell shrinkage, cell cycle arrest, mitochondrial clearance, adipogenesis, and ISC integrity maintenance and promotes neuronal health and longevity	[109, 121, 135–137]
ATG8 or LC3	Cargo selection	Maintains tissue homeostasis	[138]	LAP, apoptosis, virus replication, cancer cell survival, lysosome biogenesis, and exocytosis	[68, 120, 139–141]
ATG9	Initiation	Pathogenesis of POI	[142]	Maintains lysosomal degradation, axonal degeneration, STING, and TBK1 assembly	[143, 144]
ATG10	Elongation	Not known	-	Apoptosis, deficiency leads to ALS and FTD molecular defects, lysosomal degradation, and suppression of HCV replication	[145–147]
ATG12	Elongation	Mitochondrial homeostasis, cell death, antiviral immune responses, osteoclast secretion, and pathogen control	[119, 148, 149]	Endosomal trafficking, mitochondrial apoptosis, and endosome to lysosome trafficking	[74, 150, 151]
ATG13	Initiation	Cell cycle progression	[152]	Control virus replication and cardiac development	[153, 154]
ATG14	Nucleation	Autophagosome–endolysosome fusion	[155]	Autophagic cell death	[156]
ATG16l1	Elongation	Urothelial vesicle trafficking	[157, 158]	Apoptosis	[120, 159]
ATG18	Elongation	Programmed cell size reduction	[109]	Neural homeostasis	[160]
ATG101	Initiation	Maintaining respiratory function	[161]	Not known	-
FIP200	Initiation	Maintaining respiratory function	[161]	Control virus replication	[153]
VPS15	Nucleation	Not known	-	Skeletal muscle function, endocytosis, and neuronal migration	[130, 162, 163]
VPS34	Nucleation	T-cell homeostasis	[164]	Endocytosis, receptor degradation, and cytokinesis	[130]

LAP: LC3-associated phagocytosis; iDISC: intracellular death-inducing signaling complex; Rf: reference(s); PAS: preautophagosomal structure; ISC: intestinal stem cell; STING: stimulator of IFN genes; TBK1: TANK-binding kinase 1; POI: primary ovarian insufficiency; FTD: frontotemporal dementia; ALS: amyotrophic lateral sclerosis; HCV: hepatitis C virus; VPS34: vacuolar protein sorting 34.

**Table 2:** Autophagy defects during hematopoietic differentiation in mice.

Model system	Target cells	Hematopoietic outcomes	Rf.
Fip200^flox/flox^ × Tie2-Cre	HSCs	Myeloproliferation, HSC apoptosis, and severe anemia.	[167]
Atg5^flox/flox^ × Vav-Cre		Lymphopenia, anemia, accumulation of monocytes, macrophages, and neutrophils.	[200]
Atg12^flox/flox^ × Mx1-cre		Increased apoptosis in HSCs and loss of HSCs.	[201]
Atg7^flox/flox^ × Vav-Cre		Loss of HSC function, severe myeloproliferation, defective megakaryocyte homeostasis, platelet aggregation, apoptosis in bone marrow, defective HSPC quiescence, and cell cycle arrest.	[165, 202, 203]

Atg5^flox/flox^ × CD19-Cre	B cells	Defective antibody responses in B cells, increased cell death in BM, depletion in B-1 B cells, defective B cell homeostasis, EMH, and anemia.	[183, 184, 187]
Atg5^flox/−^ × CD21-Cre		Decreased T1 B cells and follicular B-cell numbers, reduced B-1a and B-2 B-cell proportion.	[185]

Atg5^flox/flox^ × Gzmb-Cre Atg7^flox/flox^ × Gzmb-Cre	T cells	Pathogen infection, loss of memory T cell function.	[197]
Atg7^flox/flox^ × CD4-Cre		Loss of iNKT in lymphoid organs, lymphopenia, severely compromised CD8^+^ memory T cells.	[192]
Atg3^flox/flox^ × Lck-Cre		Decreased T cell numbers.	[195]
Atg5^flox/flox^ × Cre-ER^T2^		Loss of CD8^+^ T cells, a severe reduction in lymphoid-specific memory T cells.	[198]
Atg5^flox/flox^ × CD4-Cre, Atg7^flox/flox^ × Lck-Cre		Significant reduction in iNKT, CD4, and CD8 T cell numbers.	[193, 194]
Vps34^flox/flox^;CD4-Cre		Impaired T cell homeostasis and anemia.	[189]
Atg7^flox/flox^;CD4-Cre		Progressive anemia perturbed iNKT cell development.	[191]
Atg16l1^flox/flox^;Cd11c-^Cre^		GVHD with increased T cell proliferation.	[199]
Atg16l1^flox/flox^;CD4-Cre		Expanded T cell proliferation, impaired innate NK T lymphocyte development.	[190]

Atg3^flox/flox^ Ubc;cre-ERT2	NK cells	Loss of memory NK cells.	[204]
Atg5^flox/flox^;NKp46-Cre		Impairment in NK cell development, reduction in iNKs and mNKs in the spleen and BM.	[205]

ATG5^flox/flox^-Lyz-Cre	Macrophages	Perturbed lymphoid and myeloid cell homeostasis, altered macrophage differentiation.	[206]

Atg7^Flox/Flox^; Mx-Cre	Mast cells	Impairment of mast cell degranulation.	[207]

Vav-Cre × Atg7^flox/flox^ Cebpa-cre × Atg7^flox/flox^ Mx1-cre × Atg5^flox/flox^	Neutrophils and Eosinophils	Impaired neutrophil differentiation.	[208]
Atg7^flox/flox^;Lyz2-Cr		Eosinophilic inflammation.	[209]
Atg7^flox/flox^;LysM-cre		Reduced neutrophil degranulation, increased circulating neutrophil numbers, decreased inflammatory potential of neutrophils.	[210]

Atg5^flox/flox^;CD11c-Cre	Dendritic cells	Reduced migration of DCs.	[211]

Atg7^flox/flox^;PF4-Cre	Megakaryocytes and Platelets	Impaired thrombosis, robust bleeding, and platelet aggregation.	[212]
VPS34^flox/flox^;PF4-Cre		Impeded thrombus formation.	[213]
Atg5^flox/flox^;PF4-Cre		Delayed thrombus formation, pulmonary thrombosis, and significantly reduced platelet aggregation.	[214]

mNKs: mature natural killers; BM: bone marrow; iNKT: invariant natural killer T: EMH: extramedullary hematopoiesis; CD: cluster of differentiation; DCs: dendritic cells.
